# Identification of Desmoglein-2 as a novel target of *Helicobacter pylori* HtrA in epithelial cells

**DOI:** 10.1186/s12964-021-00788-x

**Published:** 2021-11-06

**Authors:** Sabine Bernegger, Robert Vidmar, Marko Fonovic, Gernot Posselt, Boris Turk, Silja Wessler

**Affiliations:** 1 Division of Microbiology, Department of Biosciences, Paris-Lodron University of Salzburg, Billroth Str. 11, 5020 Salzburg, Austria; 2 Department of Biochemistry and Molecular and Structural Biology, Jozef Stefan Institute, Jamova 39, 1000 Ljubljana, Slovenia; 3 Cancer Cluster Salzburg and Allergy-Cancer-BioNano Research Centre, University of Salzburg, Billrothstrasse 11, 5020 Salzburg, Austria

**Keywords:** Desmoglein-2, E-cadherin, *Helicobacter pylori*, HtrA, Protease

## Abstract

**Background:**

High temperature requirement A (HtrA) is an active serine protease secreted by the group-I carcinogen *Helicobacter pylori* (*H. pylori*). The human cell adhesion protein and tumor suppressor E-cadherin (hCdh1) expressed on the surface of gastric epithelial cells was identified as the first HtrA substrate. HtrA-mediated hCdh1 cleavage and subsequent disruption of intercellular adhesions are considered as important steps in *H. pylori* pathogenesis. In this study, we performed a proteomic profiling of *H. pylori* HtrA (HpHtrA) to decipher the complex mechanism of *H. pylori* interference with the epithelial barrier integrity.

**Results:**

Using a proteomic approach we identified human desmoglein-2 (hDsg2), neuropilin-1, ephrin-B2, and semaphorin-4D as novel extracellular HpHtrA substrates and confirmed the well characterized target hCdh1. HpHtrA-mediated hDsg2 cleavage was further analyzed by in vitro cleavage assays using recombinant proteins. In infection experiments, we demonstrated hDsg2 shedding from *H. pylori*-colonized MKN28 and NCI-N87 cells independently of pathogen-induced matrix-metalloproteases or ADAM10 and ADAM17.

**Conclusions:**

Characterizing the substrate specificity of HpHtrA revealed efficient hDsg2 cleavage underlining the importance of HpHtrA in opening intercellular junctions.

**Video Abstract**

**Supplementary Information:**

The online version contains supplementary material available at 10.1186/s12964-021-00788-x.

## Background

Epithelial cells are involved in tissue homeostasis and integrity, and also provide protective barriers for organs [1, 2]. In particular, the epithelial lining of the gastric mucosa is in direct contact with incorporated food, chemicals, and pathogens while controlling digestions and uptake of nutrients.

Different types of intercellular adhesions are described to form functional cell-to-cell connections in the epithelium. Tight junctions (TJ) segment epithelial cells into apical and basolateral regions and establish cell polarity by controlling the distribution of membrane proteins and lipids. Further, TJs form a paracellular barrier that controls transport of water, ions, and small molecules [3, 4]. They are composed of transmembrane proteins such as junctional adhesion molecules (JAMs), occludin, and claudins, which are connected to the intracellular actin cytoskeleton via zonula occludens (ZO)-1 and additional TJ complex proteins. At the basolateral side of TJs, the group of adhesion contacts is located, which can be distinguished in adherens junctions (AJs) and desmosomes. These junctions serve the mechanical coupling of adjacent cells to ensure the integrity of tissues, which are exposed to high mechanical stress. In AJs, the transmembrane protein E-cadherin (hCdh1) represents the key molecule that forms calcium-dependent homophilic *cis* and *trans* interactions of its extracellular domain between neighboring cells [5]. The cytoplasmic domain of hCdh1 is bound to armadillo repeat family proteins, such as β-catenin and p120 catenin, as well as other adaptor proteins which stabilize the AJ complex and link the intracellular domain of hCdh1 to the F-actin network [6–8]. In contrast to AJs, desmosomes are not anchored to actin filaments, but to intermediate filaments of the cytoskeleton of epithelial cells. The transmembrane proteins, which mediate contact with the neighboring cell in the extracellular space, belong to the superfamily of cadherins [9, 10], including various isoforms of human desmogleins (hDsg) 1–4 and desmocollins (hDsc) 1–3 [11]. Desmosomal cadherins are composed of four highly conserved extracellular domains (EC1-4) followed by a more variable extracellular anchoring domain, a transmembrane domain, and an intracellular anchoring domain [12]. The N-terminal domain can interact in the presence of calcium in the *cis* or *trans* position allowing adhesion between the cells. The cytoplasmic domains bind to the armadillo proteins plakoglobin (PG) and plakophilins (Pkp 1–3). These are connected to the intermediate filaments of the cytoskeleton of the cell via desmoplakin (DP) [13, 14].

The intact gastric epithelium is the primary target for *Helicobacter pylori* (*H. pylori*) in the stomach. *H. pylori* is a bacterial group-I carcinogen, which persistently colonizes the human mucosa and can induce chronic gastritis, ulceration of the stomach and duodenum, mucosa-associated lymphoid tissue (MALT) lymphoma, or gastric cancer [15, 16]. Aberrant ectodomain shedding of hCdh1 and subsequent disruption of AJ and the gastric epithelial integrity is a hallmark of *H. pylori* pathogenesis. Upon *H. pylori* infection, the expression and activity of several hCdh1 cleaving host cell proteases are induced, including members of the A disintegrin and metalloprotease (ADAM) and matrix metalloprotease (MMP) families [17]. In particular, activated MMP-3, MMP-7, or ADAM-10 can target the extracellular domain of E-cadherin on the cell surface [18, 19]. hCdh1 consists of five extracellular domains (EC1–EC5), a linker region, a transmembrane domain (TMD) and an intracellular domain (IC). Cleavage of hCdh1 by host cell proteases leads to the formation of a soluble ~ 80 kDa N-terminal fragment (NTF) consisting of the extracellular hCdh1 domain [20]. Cleavage sites were located in the EC4 domain as well as close to the transmembrane domain of hCdh1 [21, 22]. Shedding of hCdh1 in *H. pylori*-infected gastric epithelial cells was originally attributed to the activation of ADAM10 [23]. However, although host cell proteases are involved in hCdh1 shedding, we identified the *H. pylori*-secreted serine protease high temperature requirement A (HpHtrA) as the main factor responsible for hCdh1 cleavage during *H. pylori* infection [24]. Between the individual EC domains of hCdh1, the [VITA]-[VITA]-x-x-D-[DN] signature pattern serves as preferred cleavage sites for HpHtrA [25]. A similar cleavage site A^698^QPV^↓^EAG^704^ with the valine in position P1 was also found in the linker region of hCdh1 [26]. Functionally, HpHtrA cleaves the extracellular domain of hCdh1 and opens intercellular AJs [24]. HpHtrA-mediated AJ disruption allows *H. pylori* to transmigrate across the epithelial barrier, which is necessary to efficiently translocate the effector protein cytotoxin-associated gene A (CagA) into infected host cells [24, 27]. In addition to the AJ adhesion molecule hCdh1, the TJ proteins claudin-8 and occludin were shown to function as HpHtrA substrates [27] indicating that secreted HpHtrA plays a superordinated role in opening intercellular adhesions.

In this study, we treated intact gastric epithelial cells with recombinant HpHtrA and used LC–MS/MS for identification of membrane proteins released from the cell surface as described previously [28]. We identified hDsg2, neuropilin-1, ephrin-B2, and semaphorin-4D as putative novel extracellular HpHtrA substrates. Corresponding to hCdh1, HpHtrA cleaved hDsg2 to a very high extent. Using an *H. pylori* HpHtrA deletion mutant and pharmacological inhibitors against matrix metalloproteases (MMPs) or A Disintegrin And Metalloproteinase domain-containing protein 10 and 17 (ADAM10, ADAM17), we showed that HpHtrA is the protease responsible for the cleavage of hDsg2.

## Results

### HpHtrA cleaves human desmoglein-2, neuropilin-1, ephrin-B2, and semaphorin-4D as novel cell surface substrates

**Fig. 1:**
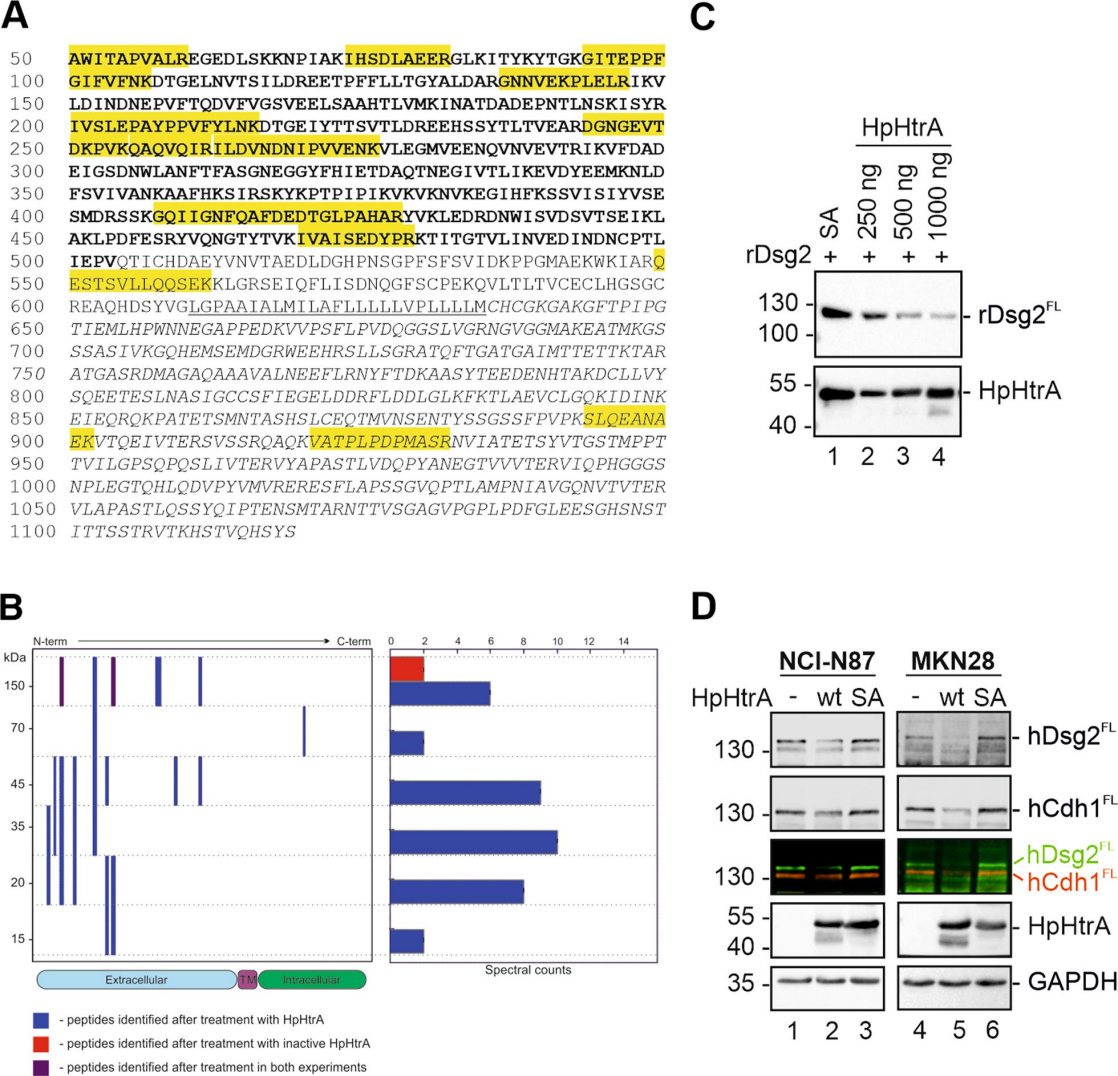
Identification of Desmoglein-2 as a new extracellular target for HpHtrA in vitro*.* **A** Peptides identified with proteomic analysis are highlighted in yellow in the hDsg2 protein sequence (UniProt Q14126), which consists of an extracellular domain (bold), a linker region followed by a transmembrane domain (underlined), and an intracellular domain (italicized). **B** Peptograph of hDsg2 peptides identified in supernatant of cells treated with HpHtrA wt and inactive mutant HpHtrA SA. Left side of the peptograph shows sequential positions of peptides identified in specific gel sections, while right side shows the number of peptide spectral counts detected in corresponding gel sections. **C** 100 ng recombinant rDsg2 were incubated with indicated amounts of recombinant HpHtrA wildtype (wt) or 1 µg inactive HpHtrA (SA) for 16 h at 37 °C. Proteins were separated by SDS-PAGE and analyzed by Western blot. rDsg2 was detected using an antibody recognizing the extracellular domain of hDsg2 (rDsg2^FL^). HpHtrA was detected by using a polyclonal HpHtrA antibody. **D** 40 µg protein lysate of NCI-N87 (lanes 1–3) or MKN-28 cells (lanes 4–6) were incubated with 250 ng HpHtrA wt or inactive HpHtrA SA for 16 h at 37 °C. Proteins were separated by SDS-PAGE and analyzed by Western blot. Full length hDsg2 (hDsg2^FL^) and full length hCdh1 (hCdh1^FL^) were detected by using antibodies recognizing the extracellular domains of hDsg2 and hCdh1. Overlay of hDsg2 (green) and hCdh1 (red) was presented to exclude possible cross reactions of the anti-hDsg2 and anti-hCdh1 antibodies. Loading control was performed by the detection of GAPDH and HpHtrA

HpHtrA has been shown to interfere with the epithelial integrity through the cleavage of the cell adhesion protein hCdh1 [24], claudin-8, and occludin [27]. In this study, we used a proteomic approach to identify novel HpHtrA substrates with putative functions in bacterial pathogenesis. Intact MKN28 cells were treated with recombinant HpHtrA and released membrane proteins were analyzed by LC–MS/MS. We identified the membrane proteins desmoglein 2 (hDsg2), neuropilin 1 (Nrp1), ephrin B2 (EphB2), semaphorin 4D (Sema4D), and E-cadherin (hCdh1), which showed significant enrichment in signal intensity and MS/MS counts after treatment of cells with active HpHtrA wild type (wt) in comparison to control cells treated with the proteolytic inactive mutant HpHtrA S_221_A (SA). Remarkably, hDsg2 exhibited the strongest increase in MS/MS count and signal intensity among all identified membrane protein ectodomains and was also higher than of the well-known substrate hCdh1 (Table 1). Most of the identified hDsg2 peptides were localized in the extracellular region of the protein, which indicates that proteolytical cleavage occurred at the cell surface (Fig. 1A, B).

**Table 1:** Shedding of surface proteins from MKN28 cells by HpHtrA

Protein^1^	Peptides (ctrl^2^)	Peptides (HpHtrA)	MS/MS counts (ctrl)	MS/MS counts (HpHtrA)	LFQ intensity (× 10^6^) (ctrl)	LFQ intensity (× 10^6^) (HpHtrA)
Desmoglein-2	2	13	1	43	37	966
Neuropilin-1	0	3	0	4	0	43
Ephrin-B2	1	3	1	5	0	30
Semaphorin-4D	0	2	0	2	0	12
E-cadherin	7	9	10	25	317	740

^1^Table presents the list of membrane proteins, which were found enriched in the supernatants of HpHtrA-treated cells and cells treated with the inactive mutant of HtrA SA (ctrl). For each identified protein, the number if identified peptides, number of identified MS/MS spectra and their intensity are shown

^2^Abbreviations: ctrl, control, wild type; MS, mass spectrometry; LFQ, label free quantification

To validate hDsg2 as potential target of HpHtrA, we performed in vitro cleavage assays using 100 ng recombinant hDsg2 (rDsg2) and increasing amounts of active HpHtrA or its isogenic inactive mutant HpHtrA SA. Full length rDsg2 (rDsg2^FL^) was detected by Western blot using an antibody recognizing the extracellular domain of hDsg2. 250 ng active HpHtrA was sufficient to partially cleave rDsg2, which can be observed as a drastic loss of rDsg2^FL^. This effect was enhanced using increasing amounts of HpHtrA (Fig. 1C, lanes 2–4). Incubation of rDsg2 with 1 µg inactive HpHtrA SA served as negative control (Fig. 1C, lane 1). To further confirm cleavage of native hDsg2^FL^, we also performed in vitro cleavage assays with whole cell lysates of the human gastric epithelial cell lines NCI-N87 (Fig. 1D, lanes 1–3) and MKN28 (Fig. 1D, lanes 4–6). NCI-N87 and MKN28 cells were disrupted by sonification in cleavage buffer, incubated with active HpHtrA wt or inactive HpHtrA SA and analyzed by Western Blot for hDsg2 and hCdh1. In agreement with the in vitro cleavage assays (Fig. 1C), a loss of the cellular 150 kDa hDsg2^FL^ was observed after treatment with HpHtrA wt in both cell lines (Fig. 1D, lane 2 and 5), while incubation with HpHtrA SA did not affect hDsg2^FL^ compared to the untreated sample (Fig. 1D, compare lane 3 and 6 with lane 1 and 4). As a control, hCdh1 was detected at 130 kDa using an antibody recognizing the EC5 domain (Fig. 1D). Similar to the detection of hDsg2, a loss of hCdh1 was only observed in the sample treated with HpHtrA wt, while the amounts of hCdh1 in the untreated sample and HpHtrA SA treated sample did not differ (Fig. 1D). To demonstrate specific recognition and to exclude possible cross reactivity of the antibodies, hDsg2 and hCdh1 were detected simultaneously with specific secondary antibodies coupled to different infrared dyes (Fig. 1D).

### *H. pylori-*induced desmoglein-2 cleavage on the surface of gastric epithelial cells is HpHtrA-dependent

**Fig. 2:**
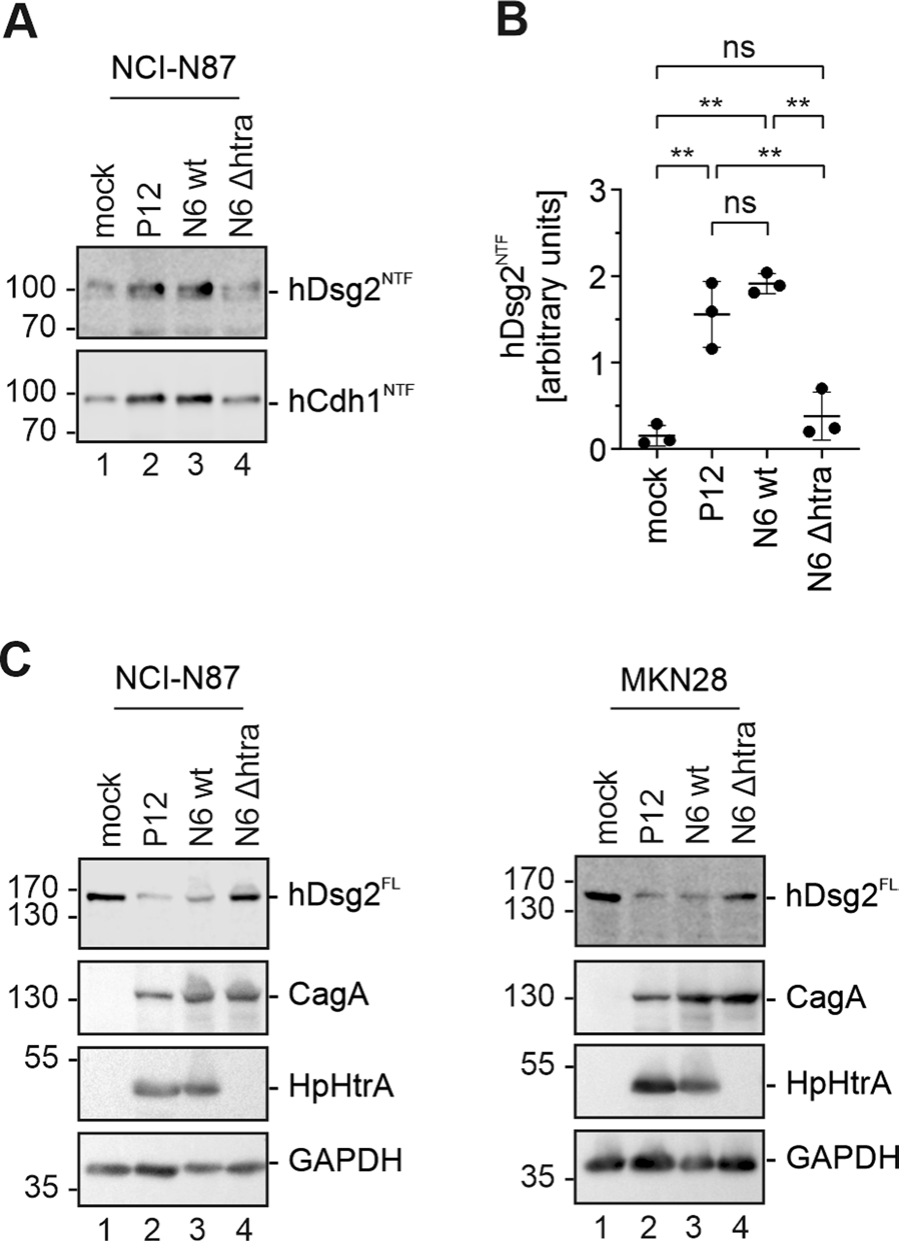
*H. pylori*-mediated Desmoglein-2 during infection of gastric epithelial cells is dependent on HtrA. Gastric epithelial cells were infected with *H. pylori* strains P12 wt, N6 wt and N6 *ΔhtrA* for 24 h or left uninfected (mock). **A** Supernatants of infected NCI-N87 cells were analyzed by Western blotting for soluble N-terminal hDsg2 (hDsg2^NTF^) and hCdh1 (hCdh1^NTF^) cleavage fragments using antibodies recognizing the extracellular domains of hDsg2 and hCdh1. **B** The amount of soluble hDsg2^NTF^ in supernatants was quantified and data are presented as mean values of hDsg2^NTF^ ± SD (n = 3). Asterisks indicate statistically significant differences (**p < 0.01; ns, not significant). **C** Whole cell lysates of infected NCI-N87 cells (left panel) and infected MKN28 cells (right panel) were analyzed by Western blotting for full length hDsg2 (hDsg2^FL^) using an antibody recognizing the intracellular domains of hDsg2. GAPDH, CagA and HpHtrA were detected as controls

The in vitro cleavage assays showed that HpHtrA directly cleaves hDsg2, which confirmed the results of the proteomic profiling of HpHtrA sheddase activity. As a next step we investigated hDsg2 shedding during infection of gastric epithelial cells with *H. pylori*. Therefore, NCI-N87 cells were colonized for 24 h with *H. pylori* strains P12 wt and N6 wt as well as the isogenic HtrA knock-out strain N6 *ΔhtrA*, which was recently published and allows to study the role of HpHtrA in *H. pylori* pathogenesis [29]. After infection of NCI-N87 cells with *H. pylori* P12 wt and N6 wt, a significant increase of a soluble N-terminal 100 kDa hDsg2 fragment (hDsg2^NTF^) in the cell culture supernatant was detected (Fig. 2A). While colonization with the HpHtrA expressing *H. pylori* strains led to an increased hDsg2 shedding, infection with the N6 *Δhtra* strain did not induce the formation of the hDsg2^NTF^ above the levels observed in uninfected cells. As a control, shedding of hCdh1 upon infection with *H. pylori* was detected (Fig. 2A). Quantification of signals detected by Western blotting from three independent experiments revealed a significant increase of the amount of the soluble extracellular hDsg2 domain in supernatants of infected cells, which was significantly different from the experiments using the HtrA-negative N6 strain (Fig. 2B). We then investigated the amount of full length hDg2 in whole cell lysates of *H. pylori* infected NCI-N87 and MKN28 cells (Fig. 2C). Correspondingly, the amount of full length hDsg2 (hDsg2^FL^) was only decreased in *H. pylori* P12 wt and N6 wt infected cells, while infection with *H. pylori* N6 *Δhtra* did not affect the amount of hDsg2^FL^ compared to the uninfected cells, underlining that hDsg2 shedding on gastric epithelial cells is HpHtrA-dependent. Detection of HpHtrA was performed to verify *H. pylori* strains and CagA and GAPDH served as infection and loading control (Fig. 2C).

**Fig. 3:**
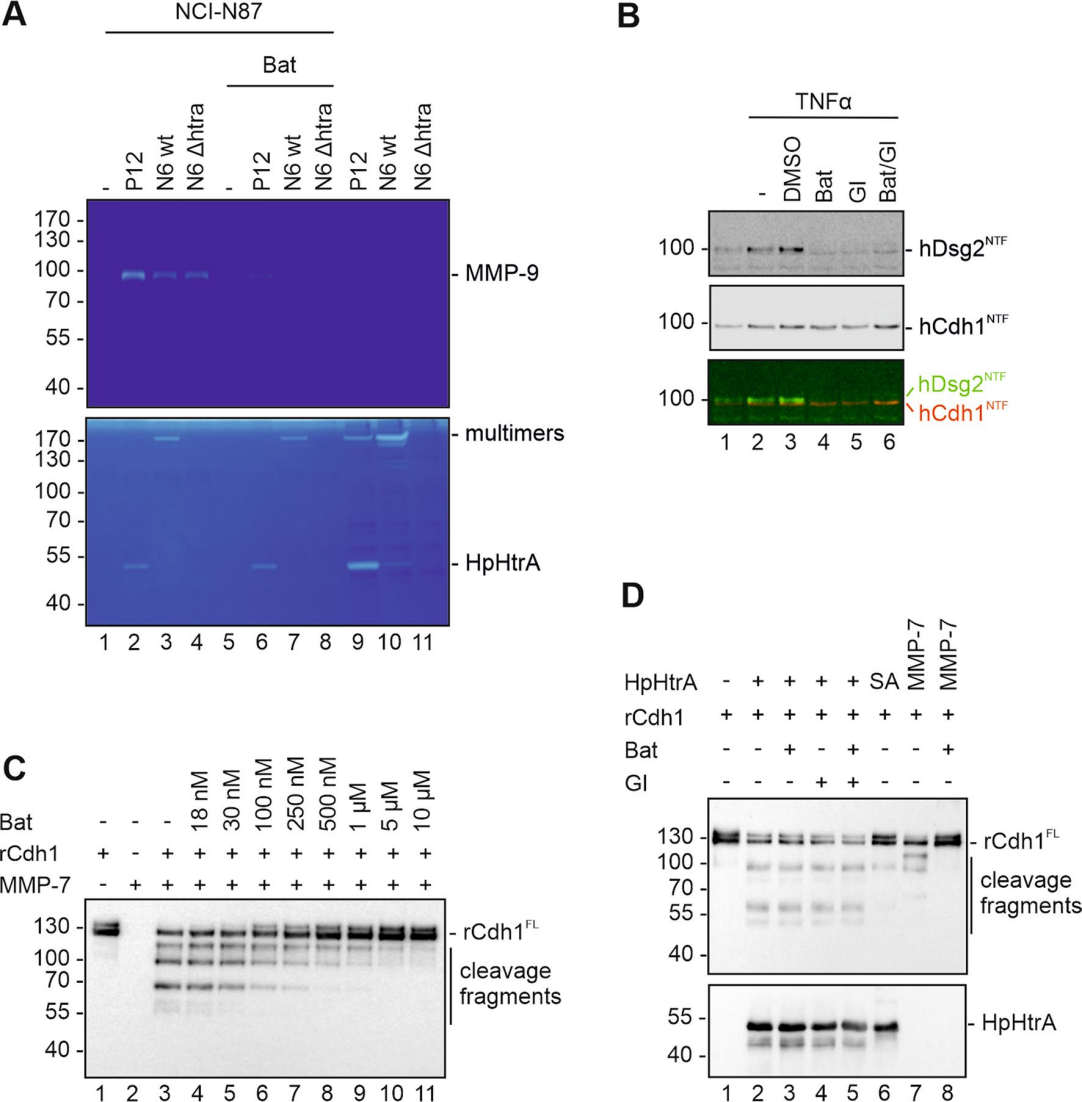
MMP and ADAM10 inhibitors do not block HpHtrA activity. **A** NCI-N87 cells were infected with *H. pylori* strains P12 wt, N6 wt and N6 *ΔhtrA* for 24 h. Where indicated, cells were treated with the broad range MMP inhibitor Batimastat (Bat) and ADAM10 inhibitor GI254023X (GI). Supernatants of infected cells were analyzed by gelatin zymography (upper panel) for proteolytic activity of activated MMPs and by casein zymography (lower panel) for proteolytic activity of secreted HpHtrA from *H. pylori* P12 wt and N6 wt. As a control, 10 µg of bacterial lysates of *H. pylori* P12 wt, N6 wt and N6 *ΔhtrA* were loaded on the zymograms. **B** NCI-N87 cells were stimulated for 24 h with 50 ng/ml TNFα to induce MMP activation. Where indicated, cells were additionally treated with broad range MMP inhibitor Batimastat (Bat), ADAM10/17 inhibitor GI254023X (GI) or a combination of both (Bat/GI). As a control, cells were incubated with 0.1% of the solvent DMSO. Supernatants of NCI-N87 cells were analyzed by Western blot for N-terminal fragments of hDsg2 (Dsg2^NTF^) and hCdh1 (Cdh1^NTF^) using antibodies recognizing the extracellular domains of hDsg2 and hCdh1. The overlay of hDsg2 (green) and hCdh1 (red) is presented to exclude possible antibody cross reactions. **C** 50 ng rCdh1 were incubated with 100 ng recombinant human MMP-7 for 16 h at 37 °C. As indicated, increasing concentrations of the broad range MMP inhibitor Batimastat (Bat) were added. Proteins were separated by SDS-PAGE and analyzed by Western blot. Full length rCdh1 (rCdh1^FL^) and cleavage fragments were detected using an antibody recognizing the extracellular domain of hCdh1. **D** 50 ng rCdh1 was incubated with 250 ng HpHtrA wt, inactive mutant (SA) or 100 ng active MMP-7 for 16 h at 37 °C. Where indicated broad range MMP inhibitor Batimastat (Bat), ADAM10 inhibitor GI254023X (GI) or a combination of both (Bat/GI) was added. Proteins were separated by SDS-PAGE and analyzed by Western blot. Full length rCdh1 (rCdh1^FL^) and cleavage fragments were detected using an antibody recognizing the extracellular domain of hCdh1. HpHtrA was detected with a polyclonal HpHtrA antibody

Shedding of both, hDsg2 and hCdh1 can also involve host cell proteases such as matrix metalloproteases (MMPs) and ADAM10/17, which are activated during inflammatory processes [30]. MMPs and ADAM10/17 have been shown to be activated in *H. pylori* infected epithelial cells [19, 31, 32]. To demonstrate MMP activation in *H. pylori* infections, we representatively detected active MMP-9 in NCI-N87 cells, which can be monitored by gelatin zymography. Infection with *H. pylori* led to a strong induction of MMP-9 activity as shown by the proteolytic 90 kDa band in supernatants of infected cells (Fig. 3A). Importantly, activation of MMP-9 was induced upon infection with the three different *H. pylori* strains independently of HpHtrA expression. To investigate whether MMPs and ADAM10/17 contribute to hDsg2 shedding during *H. pylori* infection, we used the broad range MMP inhibitor Batimastat and the ADAM10/17 inhibitor GI254023X. Pretreatment of infected cells with Batimastat strongly reduced the activation of MMP-9 after *H. pylori* infection (Fig. 3A, lines 6–8) demonstrating the efficacy of the MMP inhibitor. To exclude the possibility that the inhibitors interfere with HpHtrA activity, we also tested the same samples in a casein zymography and found unaltered proteolytic activities in the supernatants of *H. pylori* P12 wt and N6 wt (Fig. 3A, lanes 2, 3, 6 and 7) as well as in bacterial lysates of *H. pylori* P12 wt and N6 wt (Fig. 3A, lanes 9 and 10). Signals were detected at ~ 50 kDa and > 170 kDa, corresponding to HpHtrA monomers and multimers, respectively [33, 34]. As expected, in supernatants of uninfected, *H. pylori* N6 *Δhtra*-colonized cells and in lysates of *H. pylori* N6 *Δhtra* no caseinolytic activity was observed (Fig. 3A). In order to further test the inhibitory effect of Batimastat as well as GI254023X on hDsg2 cleavage in cell culture experiments, NCI-N87 cells were stimulated with the pro-inflammatory cytokine TNFα, a known activator of MMP-9 and ADAM10 [30], but also MMP-1, MMP-3, MMP-10 and MMP-12 [30, 35, 36]. As expected, treatment with TNFα led to induction of the soluble hDsg2^NTF^ fragments in cell culture supernatants (Fig. 3B, lane 2). Control treatment of cells with 0.1% DMSO did not affect hDsg2 shedding (Fig. 3B, lane 3). In fact, pretreatment of cells with Batimastat, GI254023X or a combination of both completely abolished the TNFα-mediated formation of the hDsg2^NTF^ as reported by Kamekura and colleagues [30], but not hCdh1 shedding (Fig. 3B, lanes 4–6) suggesting that TNFα can activate additional hCdh1 proteases. Further, cleavage of recombinant hCdh1 (rCdh1) by human MMP7 was efficiently inhibited by Batimastat in a concentration dependent manner (Fig. 3C). Importantly, the ability of recombinant HpHtrA to cleave rCdh1 was not affected in the presence of Batimastat and/or GI254023X compared to the untreated control (Fig. 3D, lanes 2–5), while the inactive HpHtrA SA mutant did not target hCdh1 (Fig. 3D, lane 6). Again, the proteolytic activity of recombinant MMP7 was completely abolished by Batimastat (lane 7–8). In conclusion, Batimastat and GI254023X potently inhibited the activity of MMPs as well as ADAM10/17 upon stimulation with *H. pylori* infection or TNFα treatment, but did not affect the activity of HpHtrA.

**Fig. 4:**
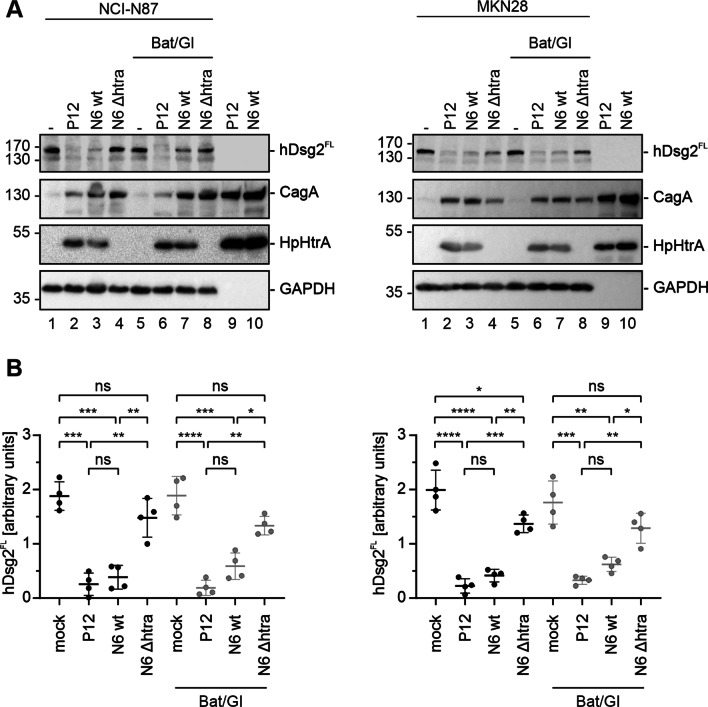
HpHtrA cleaves Desmoglein-2 on the cell surface of gastric epithelial cells independently of MMPs and ADAM10. NCI-N87 and MKN28 cells were infected with *H. pylori* P12 wt, N6 wt and N6 *ΔhtrA* for 24 h or left uninfected (mock). Where indicated, cells were treated with broad range MMP inhibitor Batimastat and ADAM10 inhibitor GI254023X (Bat/GI). **A** Whole cell lysates of infected NCI-N87 (left panel) and MKN28 cells (right panel) were analyzed by Western blot for full length hDsg2 (hDsg2^FL^). CagA, HpHtrA, and GAPDH were detected as loading controls. **B** Full length hDsg2 (hDsg2^FL^) of infected NCI-N87 cells (left panel) and infected MKN28 cells (right panel) was quantified and normalized to respective GAPDH loading controls. Data represent mean values ± S.D (n = 4). Asterisks indicate statistically significant differences (*p < 0.05; **p < 0.01; ***p < 0.001; ****p < 0.0001; ns, not significant)

After the demonstration of the functionality of inhibitors, we investigated the implication of host cell proteases in hDsg2 cleavage in *H. pylori*-infected gastric epithelial cells. Neither in NCI-N87 nor MKN28 cells, *H. pylori*-induced hDsg2 cleavage was blocked by the MMP and ADAM10/17 inhibitors, while deletion of HtrA in *H. pylori* clearly diminished hDsg2 shedding. Detection of HpHtrA was performed to verify *H. pylori* strains and CagA and GAPDH served as infection and loading control (Fig. 4A). The amount of hDsg2^FL^ in whole cell lysates from infected and inhibitor treated cells was quantified from Western blots obtained from four independent experiments (Fig. 4B). These data unequivocally demonstrate that hDsg2 and hCdh1 are directly cleaved by HpHtrA independently of *H. pylori*-induced host proteases. In summary, we identified hDsg2 as a new target for HpHtrA on the surface of gastric epithelial cells, suggesting that *H. pylori*-secreted HtrA mainly functions in the disruption of intercellular adhesions.

## Discussion

Persistent infections with *H. pylori* are the leading cause for development of gastric cancer, which is associated with one of the highest cancer-related mortality rates. Transmigration across the polarized gastric epithelium represents a major step in *H. pylori* induced pathogenesis [37, 38]. In the last years, it became evident that the secreted serine protease HpHtrA is crucially important for the bacterial passage from the apical to the basal side of the gastric epithelium, as it directly cleaves the adhesion protein and tumor suppressor hCdh1 [24, 25, 27, 39]. In fact, *H. pylori*-mediated ectodomain shedding of hCdh1 enables the contact of *H. pylori* to β_1_-integrin at the basolateral domain of the epithelial cell and facilitates translocation of the oncogenic virulence factor CagA into the host cell cytoplasm [27]. Thus, HpHtrA-mediated cleavage of hCdh1 does not only permit the passage of *H. pylori* across the epithelium, but likely plays a role in CagA-associated carcinogenesis. In this report, we identified the desmosomal cadherin hDsg2 as a new target protein for HpHtrA, suggesting that the main function of HpHtrA is the disruption of intercellular adhesions in the *H. pylori*-colonized gastric epithelium.

The AJ and TJ proteins hCdh1, occludin and claudin-8 have been previously described as HpHtrA substrates [24, 27]. In addition to hCdh1, Nrp1, EphB2, Sema4D, and hDsg2 were identified as further extracellular HpHtrA substrates in our study exhibiting the highest signal intensity upon HpHtrA treatment. Nrp1, EphB2, Sema4D were not further analyzed in this report; however, the finding that hDsg2 exhibited an eminently strong signal intensity among the identified HpHtrA substrates suggests an important role of hDsg2 in *H. pylori* pathogenesis. Dsg2 is a component of desmosomal junctions and highly expressed in epithelial cells and cardiomyocytes [40]. Generally, TJs and AJs are well investigated, while the function of desmosomes is less understood. Importantly, there is increasing evidence for a critical role of desmosomal adhesions and signaling in regulating intestinal barrier functions in both, health and disease. Besides its adhesive function, hDsg2 is implicated in enterocyte proliferation, barrier differentiation and induction of apoptosis [40]. Moreover, hDsg2 can act as a receptor for adenoviruses that cause respiratory and urinary tract infections [41, 42]. Bacterial pathogenesis induced by pathogenic *Escherichia coli* (EPEC, EHEC, or UPEC) pathovars can be influenced by hDsg2 as well. The type 1 pilus adhesin (FimH) expressed by UPEC binds hDsg2 to establish kidney infection in male C3H/HeN mice [43]. EPEC disrupts desmosomes, weakens cell–cell adhesion and perturbs barrier function of intestinal epithelial cells, which involves a cellular redistribution of hDsg2 during infection [44]. Pathogen-induced cleavage of desmogleins has previously been shown for hDsg1. The exfoliative toxin of *Staphylococcus aureus* was shown to cleave hDsg1, which leads to a disruption of hDsg1 function [45]. Further, *S. aureus* induced kallikreins have been demonstrated to degrade hDsg1 and filaggrin in infected keratinocytes [46]. Based on these observations we propose that hDsg2 cleavage by HpHtrA contributes to the disintegration of the gastric epithelial barrier in response to *H. pylori* infections.

The identified peptides derived from HpHtrA-mediated hDsg2 cleavage were mainly located in the extracellular region. Previous studies on the classical cadherin hCdh1 identified the Ca^2+^ binding sites as signature sites for HpHtrA [39]. Since hDsg2 also belongs to the family of Ca^2+^-dependent cadherins and shares structural components with hCdh1, it is tempting to speculate that HpHtrA targets similar signature sites in hDsg2, which are highly conserved between hCdh1 and desmosomal cadherins [11]. In our recent work, we have shown an additional cleavage site in hCdh1 in the linker region between the EC5 domain and the transmembrane domain [26]. We assume that this site is the only accessible cleavage site for HpHtrA during infection as the signature sites identified in in vitro settings are covered by the homophilic interaction between the extracellular domains of hCdh1 within intercellular adhesion complexes [25, 39]. This might explain why we observe a stable extracellular fragment derived from hCdh1 and hDsg2 during infection, which is in contrast to the results obtained in in vitro cleavage experiments [25]. Further, we can exclude a relevant contribution to hDsg2 shedding by host proteases, such as MMPs or ADAM10 and ADAM17, which have been previously suggested to shed hDsg2. Broad spectrum protease inhibitors and the use of HpHtrA-knock out mutants, allow us to pinpoint HpHtrA as the hDsg2-cleavingprotease in *H. pylori* infection.

## Conclusions

Desmosomes are important intercellular adhesion complexes between neighboring cells that link cell–cell contacts to the intermediate filament cytoskeleton in epithelia exposed to high mechanical stress. Downregulation of hDsg expression, mutations in hDsg genes, abnormal ectodomain shedding of hDsg by host proteases or blockage of the extracellular domain interaction through autoantibodies were previously shown to impair desmosome function in different tissues and result in severe disease [14, 47]. Until now, hDsg2 has not been reported as a direct target for bacterial pathogens in the gastrointestinal tract. In this study, we demonstrate for the first time that *H. pylori*-secreted HtrA directly cleaves hDsg2 on the surface of infected gastric epithelial cells. This implies that in addition to TJs and AJs, *H. pylori* HtrA targets also the integrity of desmosomes. Therefore, we conclude that *H. pylori* secretes HpHtrA to disrupt all three levels of intercellular adhesions (TJs, AJs, and hemidesmosomes) to obtain access to basolateral regions of the gastric epithelium where it can promote pathogenesis, since loss of function of both, hDsg2 and hCdh1, has been implicated in the development of gastric cancer [48, 49].

## Methods

### Cell culture and infection experiments

The gastric epithelial cell lines NCI-N87 and MKN28 were grown in RPMI-1640 (Sigma Aldrich, Vienna, Austria) supplemented with 10% FCS (Biowest, Vienna, Austria) and 1% L-glutamine (Biowest, Vienna, Austria) in a humidified atmosphere at 37 °C and 5% CO_2_. For infection experiments, cells were seeded in 6-well plates five days prior to infection. *H. pylori* strains P12 wild type (wt), N6 wt and N6 *ΔhtrA* [29] (N6 strains were a kind gift from Joanna Skorko-Glonek, Gdansk, Poland) were cultured on GC agar plates containing 10% horse serum (Biowest, Vienna, Austria) under microaerophilic conditions at 37 °C for 16 h before infection. Cells were starved 60 min prior to infection or treated with 10 µM GI254023X (CAS 260264–93-5, blocks ADAM10 and ADAM17, IC_50_ 5.3 nM and 541 nM, respectively; Sigma-Aldrich, Vienna Austria) and 20 µM Batimastat (CAS 130370-60-4, blocks metalloproteinases including MMP-1, MMP-2, MMP-3, MMP-7, MMP-9, ΔMT1, ADAM8, and ADAM17/TACE, IC_50_ = 3, 4, 20, 6, 4, 2.08, 51.3, and 19 nM, respectively, Merck, Darmstadt, Germany). NCI-N87 and MKN28 cells were infected with *H. pylori* P12 wt, N6 wt and N6 *ΔhtrA* at a MOI (multiplicity of infection) of 50 for 24 h or left untreated (mock). For activation of MMPs and ADAM proteins, NCI-N87 cells were seeded into 6-well plates and cultivated for five days. Cells were treated with 50 ng/µl TNFα (Cell Signaling Technology, Frankfurt, Germany) for 24 h to stimulate activation of MMP-9 and ADAM10 [30]. Simultaneously, cells were treated with 10 µM GI254023X, 20 µM Batimastat or a combination of both inhibitors. Untreated cells and cells treated with 0.1% DMSO served as control. For preparation of whole cell lysates, gastric epithelial cells were harvested in lysis buffer (20 mM Tris pH 7.5, 1 mM EDTA, 100 mM NaCl, 1% Triton X-100, 0.5% DOC, 0.1% SDS, 0.5% NP-40) supplemented with 1× PIT (protease inhibitor cocktail cOmplete EDTA-free tablets (Roche, Vienna, Austria), 1 mM sodium molybdate, 20 mM sodium fluoride, 20 mM β-glycerophosphate and 1 mM sodium orthovanadate. Whole cell lysates were cleared by centrifugation at 16,000×*g* for 10 min at 4 °C. Supernatants of infected cells were collected for detection of soluble N-terminal fragments of E-cadherin (hCdh1) and Desmoglein-2 (hDsg2), as well as for zymography. Infection experiments of NCI-N87 cells and MKN28 cells with *H. pylori* were repeated four times. Experiments for induction of MMP and ADAM10 activation were repeated two times.

### Recombinant proteins

Recombinant human Desmoglein-2 (rDsg2, A49-G608, accession no. CAA81226) was purchased from R&D Systems (Abingdon, United Kingdom). According to the manufacturer´s instructions, lyophilized rDsg2 was reconstituted in sterile Dulbecco’s phosphate buffered saline (PBS) containing CaCl_2_ and MgCl_2_ (Sigma-Aldrich, Vienna, Austria) to a final concentration of 100 ng/µl. Recombinant human E-cadherin (rCdh1, D155-I707, accession no. NP_004351) was obtained from Sino Biologicals (Vienna, Austria). Lyophilized rCdh1 was reconstituted in sterile water according to the manufacturer’s instructions at a final concentration of 250 ng/µl. Recombinant, active human MMP-7 (EC 3.4.24.23) was purchased from Sigma Aldrich (Vienna, Austria) at concentration of 100 ng/µl. Purification of HpHtrA from *H. pylori* wild type strain Hp26695 (HpHtrA, G18-K475, UniProt G2J5T2, EC 3.4.21.107) and its isogenic inactive mutant HpHtrA S_221_A (SA) was performed as previously described [33]. Briefly, *E. coli* BL21 transformed with an HpHtrA-GST expression vector were grown in terrific broth (TB) medium to an OD_600_ ~ 0.7. Expression of GST-HpHtrA was induced by addition of 100 µM IPTG for 3 h at 30 °C. Bacterial cells were harvested by centrifugation and lysed by sonication on ice 6 times 30 s with 50% power (Sonoplus Ultraschall Homogenisator HD270, Bandelin electronic GmbH, Berlin, Germany) in PBS. GST-tagged HtrA proteins were bound to glutathione sepharose beads (GE Healthcare Life Sciences, Vienna, Austria) and GST-tag was removed by addition of PreScission protease (GE Healthcare Life Sciences, Vienna, Austria) for 16 h at 4 °C. Recombinant HpHtrA was eluted and dialyzed against 50 mM HEPES (pH 7.4) and 150 mM NaCl. Purity of recombinant proteins is routinely checked with SDS-PAGE and staining with Coomassie Brilliant Blue G250 (Carl Roth, Karlsruhe, Germany).

### Proteomic identification of proteins shed by HpHtrA

MKN-28 cells were detached using an enzyme-free cell dissociation solution Hank's based (Millipore). Cells were washed with PBS, resuspended in 50 mM HEPES buffer (pH = 7.4) containing 1 mM EDTA and 4 µM recombinant HpHtrA wt and incubated for 1 h at 37 °C. As a negative control, cells were treated with the isogenic inactive mutant HpHtrA S_221_A (SA). After the incubation, the supernatant was collected (sample was centrifuged for 5 min at 500×*g*, supernatant was removed and centrifuged again for 5 min at full speed) and prepared for LC–MS/MS analysis as described previously [28]. LC–MS/MS analyses were performed with an EASY-nanoLC II HPLC unit (Thermo Scientific) coupled to an Orbitrap LTQ Velos mass spectrometer (Thermo Scientific). The peptide sample was first loaded on a C18 trapping column (Proxeon EASY-ColumnTM, 2 cm (length), 100 μm internal diameter, 5 μm 120 Å, C18-A1 beads) and then separated on a 10 cm long C18 PicoFrit™ AQUASIL analytical column, (75 µm internal diameter, 5 μm 100 Å, C18 beads) (New Objective) using forward flushing. Peptides were eluted with a 90 min linear gradient of 5–50% solvent B (0.1% formic acid in acetonitrile) at a flow rate of 300 nl/min. MS spectra were acquired in the Orbitrap analyzer with a mass range of 300–2000 m/z and 30,000 resolution. MS/MS spectra were obtained by HCD fragmentation (normalized collision energy at 35) of the nine most intense precursor ions from the full MS scan. Dynamic exclusion was enabled with repeat count of 2 and 120 s exclusion time. The database search and quantification by spectral counting were performed using the MaxQuant proteomics software (version 1.6.3.4), with imbedded Andromeda search engine [50, 51]. Search was performed against the Uniprot human protein database (20,399 sequences, download date 21.12.2018), using the trypsin cleavage specificity with maximum 2 missed cleavages. Carbamidomethylation of cysteines was set as static, while methionine oxidation and N-terminal acetylation were set as dynamic modifications. Precursor and fragment mass tolerances were set at 6 and 20 ppm. Reversed database search was performed and false discovery rate (FDR), was set at 1% for peptide and protein identifications.

### In vitro cleavage experiments, SDS-PAGE and Western Blot

For in vitro cleavage experiments, 100 ng rDsg2 was incubated with 250 ng, 500 ng or 1 µg recombinant HpHtrA wild type (wt), 1 µg inactive HpHtrA S_221_A (SA) or 100 ng recombinant active human MMP-7 in 20 µl 50 mM HEPES (pH 7.4) and 150 mM NaCl for 16 h at 37 °C. 50 ng rCdh1 was incubated with 250 ng recombinant HpHtrA wt, inactive HpHtrA SA or 100 ng recombinant human MMP-7 in 20 µl 50 mM HEPES (pH 7.4) and 150 mM NaCl for 16 h at 37 °C. Where indicated, the broad range MMP inhibitor Batimastat or ADAM10 inhibitor GI254023X was added. For in vitro cleavage experiments using NCI-N87 and MKN28 cell lysates, cells were seeded into 6-well plates and grown for five days. After harvesting in 50 mM HEPES (pH 7.4) and 150 mM NaCl, cells were lysed by pulling 10 times through a 27G needle (B. Braun, Maria Enzersdorf, Austria) and additionally by sonication on ice 6 times 30 s with 50% power (Sonoplus Ultraschall Homogenisator HD270, Bandelin electronic GmbH, Berlin, Germany). Lysates were cleared by centrifugation at 16,000×*g* for 10 min at 4 °C. 40 µg NCI-N87 or MKN28 lysate was incubated with 250 ng recombinant HpHtrA wt or inactive HpHtrA SA in 20 µl 50 mM HEPES (pH 7.4) and 150 mM NaCl for 16 h at 37 °C. In vitro cleavage experiments were repeated three times. Protein samples obtained from infection or in vitro cleavage experiments were separated by SDS-PAGE and blotted on a nitrocellulose membrane. For detection of hDsg2, antibodies against the extracellular domain (AH12.2, Santa Cruz Biotechnologies, Heidelberg, Germany) and the intracellular domain (F-8, Santa Cruz Biotechnologies, Heidelberg, Germany) of hDsg2 were used. hCdh1 was detected using antibodies against the extracellular domain EC5 (EP700Y, Abcam, Cambridge, UK) and the intracellular domain (24E10, Cell Signaling Technology, Frankfurt, Germany) of hCdh1. Polyclonal sera were used to detect HpHtrA and CagA. GAPDH (14C10, Cell Signaling Technology, Frankfurt, Germany) was used as a loading control for whole cell lysates. Simultaneous detection of hDsg2 and hCdh1 on the same membrane was done using secondary antibodies coupled to infrared dyes (IRDye680RD and IRDye800CW, Li-Cor Biosciences, Bad Homburg, Germany) and analyzed with the Odyssey Fc Imaging System (Li-Cor Biosciences, Bad Homburg, Germany).

### Zymography

For detection of proteolytic activity in cell culture supernatants of infected NCI-N87 cells, non-reducing sample buffer (125 mM Tris pH 6.8, 20% glycerol, 4% SDS, 0.02% bromophenol blue) was added to the supernatant and incubated for 10 min at room temperature (RT). Samples were separated with SDS-PAGE containing 0.1% gelatin (Carl Roth, Karlsruhe, Germany) or 0.1% casein (Carl Roth, Karlsruhe, Germany) as a substrate. Proteins in the gel were renatured by incubation for 2 × 30 min in 2.5% Triton X-100 and equilibrated for 30 min in developing buffer (50 mM Tris pH 7.5, 200 mM NaCl, 5 mM CaCl_2_, 0.02% Brij-35). After incubation in developing buffer for 16 h at 37 °C gels were stained with 0.5% Coomassie Blue R250 (Carl Roth, Karlsruhe, Germany).

### Statistics

Densitometric quantification of hDsg2 N-terminal fragment (hDsg2^NTF^), full length hDsg2 (hDsg2^FL^) and GAPDH was performed using ImageJ software. Signals were adjusted to average blot intensity and hDsg2^FL^ was normalized to respective GAPDH loading controls. Statistical analysis was performed with GraphPad Prism Software (Ver. 8.0.2) using two-way ANOVA and the Tukey’s multiple comparisons post hoc test to compare each mean to every other mean. Three independent experiments were analyzed for hDsg2^NTF^ and four independent experiments were analyzed for hDsg2^FL^ quantification. Significance is indicated as non-significant (ns) for *p* > 0.05, * for *p* < 0.05, ** for *p* < 0.01, *** for *p* < 0.001, **** for *p* < 0.0001.

## Data Availability

The mass spectrometry proteomics data have been deposited to the ProteomeXchange Consortium via the PRIDE (52) partner repository with the dataset identifier PXD025360.

## Abbreviations

CagA: Cytotoxin-associated gene A
hCdh1: Human E-cadherin
hDsg2: Human desmoglein 2
HpHtrA: *Helicobacter pylori* High temperature requirement A
